# Effects of pay-for-performance on prescription of hypertension drugs among public and private primary care providers in Sweden

**DOI:** 10.1007/s10754-020-09278-y

**Published:** 2020-01-20

**Authors:** Lina Maria Ellegård

**Affiliations:** Department of Economics, Lund University, P.O. Box 7082, 220 07 Lund, Sweden

**Keywords:** Pay-for-performance, Hypertension treatment, Ownership, Primary health care, D23, I11, I18, J33, H73

## Abstract

This study exploits policy reforms in Swedish primary care to examine the effect of pay-for-performance (P4P) on compliance with hypertension drug guidelines among public and private health care providers. Using provider-level outcome data for 2005–2013 from the Swedish Prescription Register, providers in regions using P4P were compared to providers in other regions in a difference-in-differences analysis. The results indicate that P4P improved guideline compliance regarding prescription of angiotensin converting enzyme inhibitors and angiotensin receptor blockers. The effect was mainly driven by private providers, suggesting that policy makers should take ownership into account when designing incentives for health care providers.

**Electronic supplementary material:**

The online version of this article (10.1007/s10754-020-09278-y) contains supplementary material, which is available to authorized users.

## Introduction

Pay-for-performance (P4P), incentives tied to performance targets, has been a popular strategy to improve care quality in many countries. The research literature indicates that incentives tied to process measures (e.g., guideline compliance or screening rates) are often associated with small improvements of the performance measure, while incentives tied to health outcomes tend to be ineffective. However, the quality of the evidence is limited and there are comparatively few studies from outside the United States or United Kingdom (Eijkenaar et al. 2013; Herck et al. 2010; Ogundeji et al. 2016). Moreover, although the importance of contextual factors has been pointed out (McDonald et al. 2009), there have been few attempts to examine how providers with different characteristics differ in their reaction to P4P. In particular, although public provision is a key feature of many healthcare systems, there is to the best of our knowledge no study of whether private and public providers respond differently to P4P. Theoretically, one may expect the power of monetary incentives to be weaker for public care providers for at least two reasons. First, public providers lack a profit-maximization motive and often face soft budget constraints (Kornai et al. 2003). Second, public employees may be more driven by intrinsic motivation for their work than private employees, suggesting that external incentives (such as P4P) may be less effective in public organizations (Frey et al. 2013; Georgellis et al. 2011). Nevertheless, there are reasons why publicly employed physicians may not be completely insensitive to P4P. The components of the reimbursement scheme for providers clearly gives a signal of what is prioritized by leading policy-makers (Bénabou and Tirole 2003; Ellingsen and Johannesson 2008). Publicly employed physicians with career concerns may therefore respond to the scheme, as it allows them to signal high ability (Holmström 1999).

This study examines whether private and public primary care providers in Sweden respond similarly to P4P for compliance with hypertension drug guidelines. The Swedish primary care setting provides an excellent ground for studying such heterogeneity, as almost half of all providers are publicly owned. A study of publicly employed primary care physicians in a Swedish region indicated that they react to financial incentives, but did not examine P4P (Dackehag and Ellegård 2019). Of the three existing studies focusing on P4P in Sweden, none has addressed the potentially different responses across public and private providers. The previous Swedish studies indicated that P4P is associated with improvements of process measures such as antibiotics guideline compliance (Ellegård et al. 2018), registrations in a diabetes quality register (Ödesjö et al. 2015), and medication reviews (Ödesjö et al. 2017), though not with intermediate outcomes connected to elderly or diabetics patients (Ödesjö et al. 2015, 2017). The similarity with the international evidence on P4P indicates that the Swedish experience may be of interest outside the study context.

A second contribution of the study is to extend the limited evidence base regarding P4P for hypertension treatments. This literature, which has focused exclusively on the US and UK, has found zero or temporary effects of P4P on hypertension-related process measures and on intermediate or final health outcomes (Doran and Fullwood 2007; Lee et al. 2011; Petersen et al. 2013; Serumaga et al. 2011; Simpson et al. 2011). A major weakness of this literature is that all studies except Petersen et al. (2013) were conducted in the UK, where all providers became subject to P4P simultaneously and there was thus no control group. The decentralized healthcare system in Sweden allows for a stronger research design, as there are 21 independent county councils each designing its own reimbursement scheme for care providers. The influence of many common confounding factors can therefore be mitigated in a difference-in-differences analysis, comparing providers in counties that introduced P4P to providers in counties that did not.

## Institutional background

### Primary care in Sweden

In Sweden, the mainly publicly financed health care system is organized by 21 county councils, which are independent and geographically defined governmental bodies. Although primary care has no formal gate-keeping function in Sweden, it is the first point of contact with care for most non-acute physical and mild mental health problems. In all county councils, primary care is mostly provided by group practices employing physicians, nurses and other staff categories such as physiotherapists and psychologists. Physicians operating solo practices are rare (Anell et al., 2012a). 29% of all (group) practices were private in 2006, a share that increased to 42% by 2013. The reason for the large increase was that all counties implemented policies stimulating private entry during the period. These reforms also removed all restrictions on patients’ choice of primary care provider, and are therefore known as the *choice reforms*. In 2007–2009, some counties implemented choice reforms voluntarily.^1^ National legislation mandated such reforms to be installed in all counties by 2010 (Dietrichson et al. 2016).

Each county council decides about its own reimbursement scheme for primary care providers. Capitation accounts for the largest part of reimbursement (60–80%), with visit fees making up most of the remaining part. P4P became a popular complementary (0–5% of revenues) reimbursement type during the past two decades. In 2012, all but one county used at least one P4P measure in primary care (Anell 2009; Anell et al. 2012b).

Drug costs are not covered by the reimbursement to care providers. In some county councils, providers have a separate drug budget that they have to balance. Elsewhere, the county council takes on the responsibility for drug costs (Granlund et al. 2006).

### P4P for hypertension drug guideline compliance

The examined P4P incentives were intended to affect physicians’ choice between two hypertension drugs: angiotensin converting enzyme (ACE) inhibitors and angiotensin receptor blockers (ARB). ACE and ARB are equally effective, but ACE is cheaper and therefore often recommended as first-line treatment, although it gives rise to a mild complication (cough) in a minority of patients (Godman et al. 2013). In 2010, the price differential fell as generic ARB became available in the Swedish market.

**Fig. 1:**
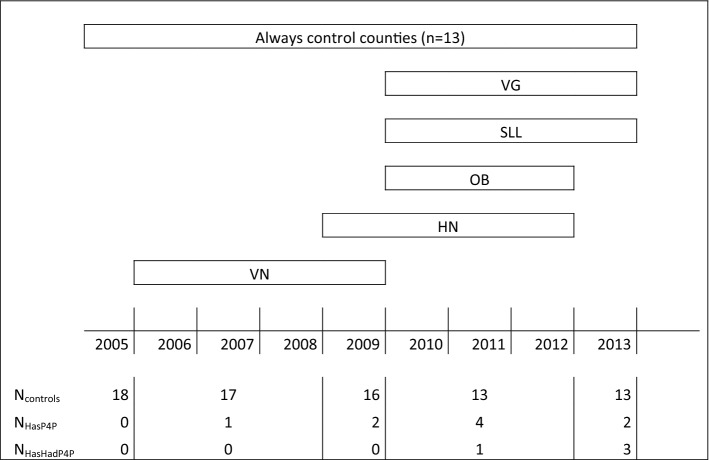
Timeline of treatment status for counties in estimation sample. *Note* The figure shows the composition of treatment and control groups over time. N_controls/HasP4P/HasHadP4P_ = number of counties that *are always in the control group*/*currently has P4P*/*have previously had P4P,* respectively, for each year

Of the eight county councils that used P4P related to hypertension drug guidelines during the study period (Table 1 and Fig. 1), six used performance targets that were directly related to ACE’s share of all ACE and ARB redemptions, with target levels around 75–80%. In the two other counties, the ACE share was incentivized indirectly, via an incentive for high compliance with all drug guidelines (i.e., not only hypertension). In all eight counties, target attainment was evaluated based on the performance of the health care practice as a whole, i.e., the P4P was a group incentive. The incentives were small, accounting for less than 1% of total revenues for an average health care practice.^2^ Notably, as the general physicians (GPs) are reimbursed by a monthly salary, the P4P brought no direct monetary benefit to GPs that did not own their practices (or a share thereof).

**Table 1:** Counties using P4P for ACE/ARB during study period. *Source*: Anell 2009; Anell et al. 2012b; county councils’ accreditation documents and personal communication. Information is available for 2005–2013

County	Years	Direct (D)/indirect (I)^b^	Target^c^
Västernorrland (VN)	2006–2009	D	2 levels: 62/73%
Skåne^a^	2009–2011	D	80%
Halland (HN)	2009–2012	D	80%
Södermanland^a^	2010–2011	D	80%
Örebro (OB)	2010–2012	D	2 levels: 76/86%
Stockholm (SLL)	2010–2013	I	80%
Västra Götaland (VG)	2010–2013	I	49–55%
Blekinge^a^	2012	D	70%

^a^Excluded from main analysis

^b^*Direct* means that the P4P target was explicitly related to the ACE share. *Indirect* means that the P4P target referred to the guideline compliance rate for *all* prescribed drugs (not only hypertension drugs)

^c^In counties with Direct targets, the target level refers to the ACE share. In counties with Indirect targets, the target level refers to the total guideline compliance rate for all drugs

## Materials and methods

### Data

#### Data sources

The analysis uses yearly data for 2005–2013 from the Swedish Prescription Register (SPR), which covers all redemptions of drugs prescribed in outpatient care. The data is aggregated at the provider level—the unit of analysis—and includes information about the county council in which the provider is located and if it is private or public.

Information about county council-level policy variables were collected by a research assistant by reviews of official documents and correspondence with county council administrators. Data on the number of GP visits per inhabitants was collected from the Swedish Association of Local Authorities and Regions.

Because the study used aggregated data, the analysis did not require an ethical permission.

#### Sample

A first sample delimitation was to include only providers that had prescribed ACE and ARB each year they appeared in the register. Further exclusions were necessary because the SPR does not reliably distinguish between prescriptions issued in primary versus secondary outpatient care. To obtain a sample including mostly primary care providers, it was recognized that, compared to the more specialized clinics in secondary care, primary care providers treat a wide spectrum of conditions, reaching from respiratory tract infections to mild mental problems (Anell et al. 2012a). Thus, a provider was included in the estimation sample if it fulfilled the following inclusion criteria:

1. The provider had issued at least one prescription each of ACE and ARB every year it appeared in the register;
2. The provider had prescribed a broad range of substances, i.e., at least one antibiotics prescription (ATC code J01) and at least one prescription of nasal preparations (R01), cough medicine (R05) antidepressants (ATC N06AA) and/or hypnotics (N05C) during the years it appeared in the register.

2202 providers fulfilled criterion 1 in 2010; of these, 1220 also fulfilled criterion 2. This is only 10 providers short of the total number of primary care providers in 2010 according to existing registers of Swedish primary care providers (SPCP) (Dietrichson et al. 2016). Providers in the eight P4P counties accounted for 61% of all providers in the estimation sample, to be compared to 63% in the SPCP. The number of private providers was higher in the estimation sample (529 vs. 470), which may due to misclassification but can also be explained by the under-coverage of private solo practices in SPCP. On balance though, the resulting estimation sample was deemed as reasonable. The Supplementary material to the paper includes a robustness check of our main model specification using an estimation sample defined by inclusion criterion 1 only.

#### Variables

The main outcome variable was ACE’s share of all ACE and ARB redemptions, i.e., the number of ACE redemptions divided by the total number of ACE and ARB redemptions. The number of ACE and ARB redemptions were also analyzed separately.

Three county council-level variables were included as covariates: a dummy indicating counties where primary care providers had budget responsibility for prescribed drugs, a dummy indicating years after the choice and entry reforms, and the number of GP visits per 1000 inhabitants in the county council.

Table 2 show definitions and summary statistics for the variables in the analysis.

**Table 2:** Summary statistics and variable definitions

	2006				2013			
	Control		P4P		Control		Ever P4P	
	Mean	SD	Mean	SD	Mean	SD	Mean	SD
*Panel A: Summary statistics*								
ACE share	0.62	0.18	0.49	0.21	0.57	0.17	0.52	0.19
ACE	1058	1089	514	799	1190	1538	763	1127
ARB	562	629	428	543	817	1066	618	843
PrivOwn	0.31	0.46	0.52	0.5	0.32	0.47	0.5	0.5
DrugBudget	0.47	0.5	0.28	0.45	0.94	0.23	0.3	0.46
GPvisits	1294	114	1543	154	1345	153	1757	309
ChoiceReform	0	0	0	0	1	0	1	0
No. providers	427		463		482		498	

Variable	Definition	Aggregation level	Type
*Panel B: Variable definitions*			
ACE share	Number of ACE redemptions/(number of ACE and ARB redemptions)	Provider (P)	Dependent variable (main)
ACE	Number of ACE redemptions	Provider	Dependent variable
ARB	Number of ARB redemptions	Provider	Dependent variable
PrivOwn	PrivOwn = 1 if privately owned PrivOwn = 0 if publicly owned or unknown ownership	Provider	Interaction variable
DrugBudget	= 1 if each care provider has responsibility for its own drug budget = 0 if care providers are not responsible for costs of prescribed drugs	County council (C)	Covariate
GPvisits	Number of GP visits per 1000 inhabitants	C	Covariate
ChoiceReform	Dummy for years after implementation of patient choice and free entry in primary care	C	Covariate

### Empirical strategy

A comparison of the ACE shares in P4P and non-P4P counties at a given point in time is unlikely to describe the effect of P4P, as their prescription cultures may differ for idiosyncratic reasons. Similarly, a simple before-after comparison of the outcomes within P4P counties would pick up not only the impact of P4P, but also the impact of all other simultaneous changes that affect the outcome variable. For instance, the first generic ARB was released during the study period (Godman et al. 2013). The implied change in relative prices may by itself have affected physicians’ propensity to prescribe ARB or ACE.

By estimating a difference-in-differences (DID) regression model, it is possible to account for time-varying confounders like the introduction generic ARB, which affected the whole country at the same time; at least to the extent that the impact of such confounders is homogenous. In a DID regression, the change in the outcome variable before and after the introduction of P4P is compared with the change over the same time period in counties that never used P4P (Imbens and Wooldridge 2009).

The difference in differences can be given a causal interpretation, under the assumption that the development of the outcome variable would have been the same if the P4P group had not changed their incentive scheme. It is not necessary that the treatment and control groups have a similar level of the outcome variable, as long as they follow the same trend (Imbens and Wooldridge 2009). The trend assumption may be more or less plausible for the different P4P counties. To prevent obvious violations of the assumption, an initial graphical analysis was performed in which the development of the ACE share in each P4P county was compared to the development in the control counties (see the supplementary material). In three P4P counties (Skåne, Södermanland and Blekinge), the trends were judged to be too dissimilar from control counties. These counties were excluded from the further analysis, leaving a distribution of treated and control counties as in Fig. 1.

#### Econometric specification

Equation 1 shows the baseline regression model:

1 $$y_{it} = \alpha + {{\upbeta }}_{1} *HasP4P_{it} + {{\upbeta }}_{2} *HasHadP4P_{it} + {\varvec{\upbeta}}_{3} *{\mathbf{X}}_{{{\mathbf{it}}}} + \varvec{\gamma t} + {{\upmu }}_{\text{i}} + \varepsilon_{it}$$

$y_{it}$ is the outcome variable of provider *i* at time *t*, $\alpha$ is a constant, ${\mathbf{X}}_{\text{it}}$ is a vector of time-varying covariates, $\varvec{t}$ is a vector of calendar year dummies,${{\upmu }}_{\text{i}}$ is a provider-specific dummy (fixed effect, FE), and $\varepsilon_{it}$ is an idiosyncratic error term. The provider FEs eliminate the influence of time-invariant unobserved heterogeneity. Notably, the provider FEs capture the influence of idiosyncratic features of each provider (e.g., organizational culture), and of county-council-specific features that affect all providers in a given county similarly. The provider FE thus account for the multi-level nature of the data. The calendar year dummies capture year-specific shocks (e.g., the introduction of generic ARB) to the extent that they had similar effects across the whole country.

*HasP4P* is a dummy indicating providers that were subject to P4P in year *t*, while *HasHadP4P* is a dummy for providers that previously had been, but were no longer, subject to P4P (cf. Table 1). The parameters of interest capture the effect of being, or having been, subject to ACE-related P4P. Notably, in any given year, there is no within-county variation in *HasP4P*_*it*_ *and HasHadP4P*_*it*_, i.e., all providers in a given county are classified either as 0 or 1. For providers in the 13 control counties, these variables equal zero in all observed years, whereas they vary over time for providers in the five analyzed P4P counties.

To analyze whether the response to P4P differed for private and public providers, the following equation was estimated:

2 $$y_{it} = \alpha + {\varvec{\upbeta}}_{1} *\varvec{P}4\varvec{P}_{{\varvec{it}}} + {{\upbeta }}_{2} *Priv_{it} + {\varvec{\upbeta}}_{3} *\varvec{P}4\varvec{P}_{{\varvec{it}}} *Priv_{it} + {\varvec{\upbeta}}_{4} *{\mathbf{X}}_{{{\mathbf{it}}}} + \varvec{\gamma t} + {{\upmu }}_{\text{i}} + \varepsilon_{it}$$

Compared to Eq. 1, the specification in Eq. 2 includes interaction terms between each provider’s ownership status (*Priv* = a dummy indicating privately owned providers) and $\varvec{P}4\varvec{P}_{{\varvec{it}}} \varvec{ }$, a vector including the variables *HasP4P* and *HasHadP4P*.

Standard errors were clustered at the county council level, as the P4P scheme was the same for all providers in a given county (Bertrand et al. 2004).

#### Sensitivity analyses

In addition to the graphical analysis, the similarity of the pre-P4P time trends were evaluated in the following model for 2005–2009:

3 $$y_{it} = \alpha + \gamma_{1} t + \gamma_{12} *P4P*t + {{\upmu }}_{\text{i}} + \varepsilon_{it}$$

In Eq. 3, $t$ is a linear time trend and the potentially differential trend in P4P counties is captured by the interaction between *t* and a dummy (*P4P*) for providers in the counties that would later adopt P4P. A large and significant interaction term would indicate that the P4P counties followed a different trend already before they implemented P4P. Notably, of the five P4P counties included in the analysis, one introduced P4P in 2006 (Västernorrland) and one in 2009 (Halland). Västernorrland was excluded from the estimations of Eq. 3, which was additionally estimated on a sample excluding observations from Halland and on a sample covering a shorter period (2005–2008). In another specification, deviations from the control group were captured by a dummy for the year before implementation of P4P. We also estimated an alternative DID specification that allowed for differential linear time trends in P4P and control counties (Bell et al. 1999; Li et al. 2014):

4 $$y_{it} = \alpha + {{\upbeta }}_{1} *HasP4P_{it} + {{\upbeta }}_{2} *HasHadP4P_{it} + {\varvec{\upbeta}}_{3} *{\mathbf{X}}_{{{\mathbf{it}}}} + \gamma_{1} t + \gamma_{12} *P4P*t + {{\upmu }}_{\text{i}} + \varepsilon_{it}$$

Here, P4P*t is the interaction between a time-invariant group variable (P4P = 1 throughout the sample period for all providers in counties that ever used P4P) and a linear time trend variable. Thus, $\gamma_{12}$ picks up the differential linear time trend—over the whole sample period—in the set of counties that ever used P4P. We also estimated a similar model using county council-specific linear trends and year dummies.

The influence of specific counties on the main results were evaluated in leave-one-out analyses, in which the preferred model (Eq. 2) was repeatedly estimated, each time dropping the observations from one of the P4P counties. The sensitivity of the preferred model was also examined by removing the covariates, excluding providers with very few redemptions, excluding controls with comparably high ACE shares, clustering standard errors at the provider level and applying the wild cluster bootstrap to check the sensitivity the for small number of clusters (Cameron and Miller 2015), and including providers fulfilling inclusion criteria 1 only.

## Results

**Fig. 2:**
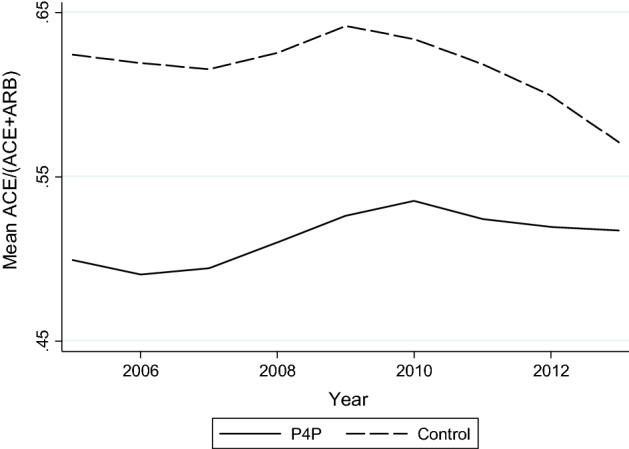
ACE share by year and P4P status. *Note*: Yearly average ACE share, calculated separately for two groups of counties: those that used P4P at some point in time (P4P) and those that never did (Control). Providers from Skåne, Södermanland and Blekinge are excluded. Within the P4P group, the identity and number of counties actually using P4P varied over the time period (see Table 1)

Although the P4P and control counties had different average ACE shares (50% vs. 62% in the early period), Fig. 2 shows that the development in the groups was similar until 2010, the year when generic ARB was introduced. For providers in the control counties, the ACE share decreased between 2009 and 2010. In the P4P counties, the ACE share did not start to decrease until the year thereafter.

Table 3 shows the DID estimates of the P4P effect. Disregarding private/public ownership (column 1), the estimated effect of P4P on the ACE share was 3 percentage points (*p* < 0.01), a 5% increase relative to the mean ACE share (56.4%). The interaction specification indicates that the P4P effect was significantly stronger—twice as large—for private providers (column 2). The changing ACE share of private providers reflected a substitution of ARB for ACE, while for public providers it was more a case of less growth of the ARB prescriptions (columns 3–5). For both provider types, the effect on the ACE share became insignificant once the incentive was removed, although one reason for the insignificance may be that only 103 observations identify these estimates.

**Table 3:** Main results

Dependent variable (y)	(1)	(2)	(3)	(4)	(5)
	ACE share	ACE share	ACE	ARB	ACE + ARB
HasP4P	0.0299***	0.0184**	151.2	44.58	195.8
	(0.00614)	(0.00753)	(139.4)	(88.49)	(226.0)
PrivOwn		− 0.00241	5.088	11.93	17.01
		(0.00658)	(58.09)	(38.42)	(93.84)
HasP4PxPrivOwn		0.0228**	− 254.3*	− 188.5**	− 442.7**
		(0.00843)	(124.1)	(79.76)	(203.6)
HasHadP4P	0.0194	0.00458	278.9*	136.7*	415.6*
	(0.0149)	(0.0124)	(141.8)	(77.17)	(215.3)
HasHadP4PxPrivOwn		0.0337	− 122.8	− 40.96	− 163.7
		(0.0306)	(87.96)	(97.93)	(168.3)
DrugBudget	0.0129	0.0133	250.4**	114.3*	364.7**
	(0.00869)	(0.00863)	(108.6)	(60.09)	(164.9)
GPvisits	3.98e−05	3.67e−05	0.0904	0.0977	0.188
	(2.60e−05)	(2.47e−05)	(0.268)	(0.164)	(0.416)
ChoiceReform	− 0.0127**	− 0.0125**	− 77.16*	− 26.91	− 104.1*
	(0.00489)	(0.00484)	(38.93)	(20.96)	(57.69)
Constant	0.502***	0.507***	85.45	12.04	97.49
	(0.0340)	(0.0327)	(374.3)	(222.9)	(570.8)
Observations	8581	8581	8581	8581	8581
R-squared	0.030	0.032	0.197	0.207	0.210
Number of providers	1029	1029	1029	1029	1029
Counties	18	18	18	18	18
Mean of y	0.564	0.564	902.2	579.5	1482
HasP4P = HasHadP4P (*p*)	0.477	0.253	0.129	0.0761	0.101
ME HasP4PxPriv (*p*)		0.000	0.292	0.0146	0.109
ME HasHadP4PxPriv (*p*)		0.218	0.241	0.338	0.200

Estimates of Eqs. 1 (column 1) and 2 (column 2–5) using the following dependent variables (y): *ACE share* = ACE’s share of all ACE and ARB redemptions in columns 1–2, *ACE* (*ARB*) = no. ACE (ARB) redemptions in column 3 (4), *ACE *+ *ARB* = total no. ACE and ARB redemptions in column 5

HasP4P = HasHadP4P (*p*) = *p* value of test of equality of coefficients. ME HasP4PxPriv (*p*) = *p* value of test of marginal effect of P4P for private providers. ME HasHadP4PxPriv (*p*) = *p* value of test of marginal effect of previously having had P4P for private providers

Robust standard errors clustered by county in parentheses. ***p < 0.01; **p < 0.05; *p < 0.1

The estimates on *PrivOwn* are also imprecise, as they are identified by a very small number of providers that have switched from public to private ownership. Giving providers responsibility for a *drug budget* did not affect the ACE share, although it was associated with increased prescription of both ACE and ARB. The county-level number of *GP visits* was not associated with the provider-level ACE share, whereas the prescription of both ACE and ARB generally decreased following a *choice reform*.

The supplementary material displays the sensitivity tests. Notably, the parallel trends assumption appeared reasonable and the main results were robust to including differential trends for P4P counties. The positive effect for private providers was robust to the sensitivity tests, although the difference between public and private was not significant when using the wild cluster bootstrap. The effect for public providers was less stable and appeared to be driven by the two largest counties (Västra Götaland and Stockholm), in which the P4P incentive was indirect (see Table 1). Notably, the positive effect on private providers remained when excluding one P4P county at a time from the analysis, which is interesting given that the targets and incentive sizes differed across counties. The results were also similar when providers that failed to meet inclusion criterion 2 were included in the sample, although the magnitude of the public–private interaction was attenuated. The attenuation was expected, given that the more comprehensive sample included a large number of providers that were not subject to P4P [i.e., outpatient secondary care clinics (Anell 2015)].

## Discussion

The results strongly suggest that private providers reacted to P4P, while public providers reacted only in a few counties. The findings are consistent with the idea that monetary incentives are of higher importance for profit-maximizing providers, and also with the idea that individuals attracted to the private sector are relatively more motivated by extrinsic rewards (Georgellis et al. 2011). At the same time, it seems premature to rule out that public providers are completely insensitive to P4P; rather, the results indicate that their reaction depend on the circumstances.

The ACE share was lower for providers in the P4P counties than for providers in the control counties, suggesting that the decision to implement the P4P policy was driven by policy-makers wanting to approach the levels of other counties. While such selectivity in the decision to adopt P4P may limit the generalizability of the findings, it is not clear in what direction the bias would go. On the one hand, it may be easier to increase the ACE share if it initially is low. On the other hand, providers in the P4P counties may have firmly grounded aversion towards ACE, making it harder to influence their prescribing decisions. Notably, because policies like this are rarely randomized, a treatment-effect-on-the-treated like the one estimated here is a relevant policy parameter.

Many of the counties using P4P for the ACE share chose to remove the indicator after a few years. One obvious reason may be that the introduction of generic ARB made the issue less important. Previous research suggests that there are many reasons why counties often choose to replace their P4P indicators: satisfaction or disappointment with achieved outcomes, experiences that the performance measure is too volatile at the health care unit level, or simply a desire to prioritize something new (Ellegård et al. 2018; Johansson Krafve 2015). In Sweden, there has also been a growing critique against the use of monetary incentives in the health care sector (SOU 2017).

The analysis has several limitations. As already discussed, it was necessary to make assumptions about which providers in the prescription registers were primary care providers. It is thus possible that some providers were misclassified, although the sensitivity analysis indicated that the consequences of the sample delimitation were small.

Another weakness of the study is that the register does not include prescriptions that the patient did not redeem. Notably though, unless patients’ redemption decisions have changed differentially over time in P4P and non-P4P counties, the difference-in-differences analysis accounts for systematic differences between P4P and non-P4P counties.

A remaining caveat is that the empirical strategy only accounts for changes over time that affected providers in all county councils similarly. For instance, if the introduction of generic ARB had different impact in different counties, the estimated “P4P” effect also includes the differential effect of price changes. However, the model mitigates this concern by controlling for whether providers were responsible for a drug budget, as such responsibility would be the only reason (apart from P4P) why providers would care about the cost of prescribed drugs. Of note, the share of control providers with a drug budget increased a lot over the sample period (Table 1), but most of the increase took place before 2010, the year when many of the treated providers became affected by P4P. Therefore, the implementation of drug budgets in control counties should by itself not be a big confounder, although we cannot completely dismiss such concerns. Likewise, in both the P4P group and the control group, there are examples of counties that introduced a choice reform in 2010 and others that had already done so. Therefore, the *choice reform* variable limits the potential disturbance from these reforms at least to some degree.

The observational study setting implies some complications. Almost all county councils experimented with different P4P indicators during the study period, and it is not possible to rule out that the effect to some degree reflects that the ACE share received *less* attention by providers in counties that just had adopted incentives for other, unrelated goals (Holmström and Milgrom 1991). In one of the P4P counties (SLL), the total number of patients prescribed ACE or ARB was also incentivized, meaning that the impact in that county might reflect a changing patient mix. Notably though, the result that private providers react to P4P was unchanged even when providers from SLL was left out of the analysis.

Notwithstanding the limitations of the study, it is interesting to note that the estimated effect of 5% was similar in magnitude to the typical estimate for process measures found in previous P4P studies (Herck et al. 2010; Ogundeji et al. 2016). The estimate was also similar in magnitude to that of a previous Swedish study that concerned incentive for compliance with antibiotics guidelines (Ellegård et al. 2018) and qualitatively similar to other findings from Sweden (Ödesjö et al. 2015, 2017). In difference to previous evidence from Sweden (Ellegård et al. 2018), but similar to findings from other contexts (Constantinou et al. 2017), the significant effect only lasted as long as the incentive was in place. However, this result is tentative, as the point estimate for private providers indicated a lasting effect and the number of private providers in this category might have been too small to detect statistical significance.

Of studies on P4P for hypertension-related performance measures, this is the first to consider the ACE share. Observational time-series studies from England and Scotland found no trend breaks around the time of P4P implementation with respect to the number of prescribed hypertension drugs or recordings of blood pressure (Lee et al. 2011; Serumaga et al. 2011; Simpson et al. 2011). A randomized control trial in the US found a temporary increase in the percentage of patients on recommended drugs when incentives targeted individual physicians, but no effect of group incentives (Petersen et al. 2013). It has been argued that the target levels in the English P4P scheme were too low to spur further improvements (Serumaga et al. 2011); by contrast, the typical target levels in Sweden (Table 1) ought to have presented a challenge for the Swedish care providers. In relation to the results from the US study, it is interesting that the Swedish incentives appear to have been effective despite that they were directed to the health center rather than to individual physicians. However, given the vast differences in terms of institutional settings and study designs, it is difficult to pinpoint the reasons why the results differ.

## Conclusions

P4P increased compliance with drug treatment guidelines for hypertension in Sweden. The effect was particularly strong for private providers, while the effect on public providers was present only in a few counties. Future research should seek to investigate the conditions under which public providers do react to monetary incentives (Dackehag and Ellegård 2019). Policymakers and researchers ought to acknowledge that the different underlying incentive structures of public and private providers may affect the effectiveness of P4P, even when little money is at stake.

## Electronic supplementary material

Below is the link to the electronic supplementary material.

Supplementary material 1 (PDF 936 kb)

## Footnotes

1 Halland in 2007, Stockholm and Västmanland in 2008, Kronoberg, Västra Götaland, Skåne, Östergötland, Uppsala in 2009.

2 In the counties with indirect targets, the size of the ACE-related incentive is impossible to calculate, as it depends on how well providers complied with other guidelines.
