# Supplementary material for: Effects of pay-for-performance on prescription of hypertension drugs among public and private primary care providers in Sweden
Source: Int J Health Econ Manag. 2020 Jan 20;20(3):215–28. doi: 10.1007/s10754-020-09278-y (PMC7426314; doi:10.1007/s10754-020-09278-y)
Supplement: Supplementary file 1 — Supplementary material 1 (PDF 936 kb) [file 10754_2020_9278_MOESM1_ESM.pdf]

Supplementary material to *Effects of pay-for-performance on prescription of hypertension drugs among public and private primary care providers in Sweden*

Development of the ACE share in the excluded counties

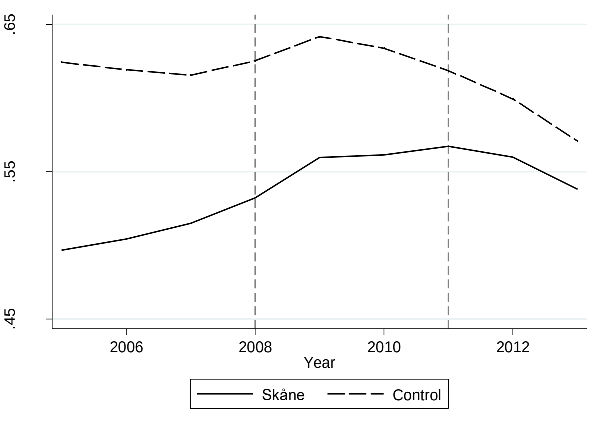

a) Skåne

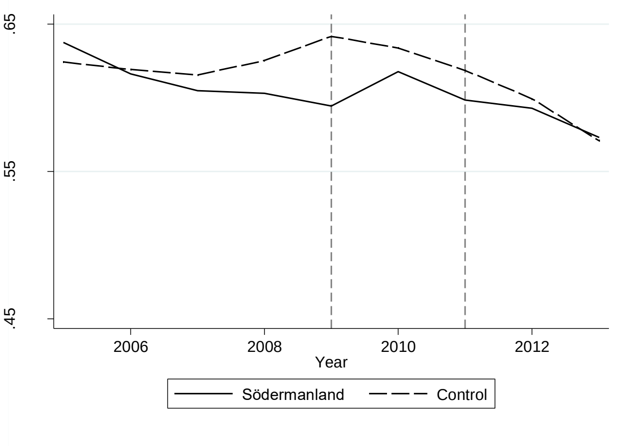

b) Södermanland

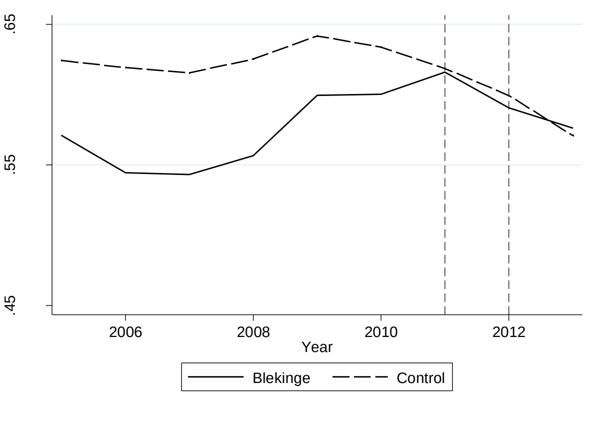

c) Blekinge

Fig S1. ACE share by year. Skåne, Södermanland and Blekinge vs. control group
The two dashed lines mark the last year before P4P was implemented and the last year P4P was in place, respectively. Skåne, Södermanland and Blekinge are not included in the main estimations.

Sensitivity tests

The first three columns of table S1 shows the pre-trend tests according to Eq. 3. Providers from Västernorrland (VN), where P4P was introduced in 2006, are excluded from columns 1-3. Eq. 3 was estimated first without providers in Halland, where P4P was introduced in 2009 (column 1), and then including these providers (column 2) and excluding 2009 (column 3). The coefficients on the interaction between being a P4P county and the linear trend variables are insignificant. Column 4 shows estimates of a variant of Eq 1 which includes a dummy for the year before a county council implements P4P ($HasP4P_{t-1}$). This specification allows us to include VN in the sample. The estimate on $HasP4P_{t-1}$ is positive but statistically insignificant.

Table S1. Pre-trend test of parallel trends

Variable	(1) ACE share	(2) ACE share	(3) ACE share		(4) ACE share
time	0.00332**	0.00329**	2.13e-05	$HasP4P_{t-1}$	0.0102
	(0.00140)	(0.00141)	(0.00177)		(0.00719)
TreatTrend	0.00382	0.00431	0.00551	HasP4P	0.0338***
	(0.00430)	(0.00400)	(0.00484)		(0.00801)
DrugBudget	0.0154**	0.0153**	0.0105	DrugBudget	0.0147
	(0.00555)	(0.00550)	(0.00685)		(0.00824)
GPvisits	-2.62e-05	-3.15e-05	-5.94e-05	GPvisits	0.00003
	(5.54e-05)	(5.49e-05)	(6.31e-05)		(0.00003)
choicereform	-0.00350	-0.00223	-0.00151	choicereform	-0.0130*
	(0.00882)	(0.00829)	(0.0108)		(0.00490)
Constant	0.580***	0.589***	0.633***	Constant	0.513***
	(0.0771)	(0.0770)	(0.0888)		(0.0405)
Observations	4,363	4,511	3,549		8,581
R-squared	0.009	0.010	0.002		0.01
Number of providers	938	969	944		1,029
Counties	16	17	17		18
Halland	No	Yes	Yes		Yes
Västernorrland	No	No	No		Yes
Years	2005-2009	2005-2009	2005-2008		2005-2013

Column 1-3 shows estimates of Eq 3. time is a linear time trend variables and TreatTrend is the interaction between time and a dummy for P4P counties. Column 4 shows a version of Eq 1 including a dummy for the year before a county implements P4P ($HasP4P_{t-1}$). Cluster-robust standard errors in parentheses. *** p<0.01, ** p<0.05, * p<0.1.

Table S2 shows the results of the preferred model (Eq. 2, col 1) and the leave-one-out specifications (col. 2-7). The results for the key variables *HasP4P* and *HasP4PxPriv* are stable unless SLL or VGR is excluded, in which case the effect for public providers disappears while the effect on private provider becomes larger. The variables indicating previous experience with P4P (HasHadP4P) are more unstable, but were also imprecisely estimated in the preferred model.

Table S2. Leave-one-out estimates of the ACE share

Excluded county:	(1) N/A	(2) VN	(3) HN	(4) OB	(5) SLL	(6) VGR	(7) SLLVG
HasP4P	0.018	0.021	0.021	0.023	0.01	0.014	-0.01
	0.008	0.008	0.007	0.006	0.012	0.008	0.004
Priv	-0.002	-0.002	-0.002	-0.002	-0.002	-0.001	-0.001
	0.007	0.007	0.007	0.007	0.009	0.007	0.009
HasP4Px Priv	0.023	0.021	0.021	0.019	0.038	0.021	0.052
	0.008	0.008	0.008	0.007	0.009	0.01	0.003
HasHad P4P	0.005	-0.003	0.013	0.004	0.002	0.005	-0.007
	0.012	0.017	0.01	0.014	0.013	0.012	0.013
HasHad P4PxPriv	0.034	0.065	0.03	-0.002	0.045	0.034	0.056
	0.031	0.008	0.04	0.032	0.029	0.031	0.024
Constant	0.507	0.51	0.506	0.52	0.54	0.498	0.582
	0.033	0.037	0.035	0.035	0.038	0.033	0.039
Observations	8,581	8,361	8,310	8,149	6,306	7,380	5,105

Column 1 shows the preferred model specification, in which 5 P4P counties are included. In columns 2-7, observations from one P4P county council at a time are excluded. VN=Västernorrland, HN=Halland, OB=Örebro, SLL=Stockholm, VGR=Västra Götaland. In column 7, both SLL and VG are excluded. The table shows coefficients and standard errors.

Table S3 shows the preferred model (column 1) along with various sensitivity tests. Column 2 shows the differential trends specification (Eq. 4), in which the P4P effect disappears for public providers but remains (although it is slightly attenuated) for private providers. Note that the significant interaction term TreatTrend does not imply a violation of the parallel trends assumption, as the trend is extended over the whole sample period in this specification. Of further note, the estimates are very similar in a specification including year fixed effects and county council-specific linear time trends.

Column 3 shows that the county level covariates are not influential for the result of the preferred model. In column 4, providers for which there were fewer than 98 redemptions of ACE and ARB (combined) are excluded from the estimation (98 is the lowest quartile of the distribution of ACE+ARB redemptions). This sample restriction mostly serves to increase the P4P effect for public providers; the total marginal effect on private providers is practically unchanged. In column 5, observations in the control group whose ACE share is higher than 70% are excluded, to check if the results are driven by the generally lower ACE-shares in P4P counties. This appears to be true to some degree, as the effect for public providers disappears (*HasP4P* becomes smaller and insignificant), while the P4P effect is attenuated (though still strongly significant) for private providers.

In column 6, standard errors are clustered at the provider level instead of the county level (Cameron and Miller 2015). The county level standard errors are sometimes, but not consistently, smaller, but the difference is always very small. When instead using the wild cluster bootstrap to draw inference, the estimate on HasP4P becomes more imprecise (p-value of bootstrap *t*-statistic=0.07), and the interaction term HasP4PxPrivOwn is no longer significant (p-value of bootstrap *t*=0.15).[1] The inference from the main model using cluster-robust standard errors is nonetheless rather robust: the 95% confidence interval for the bootstrapped interaction term does not include zero, and the significance of HasP4P increases substantially in a specification without the *PrivOwn* interaction term (p-value of bootstrap *t* for *HasP4P* =0.0028).

In column 7 we relax the second sample inclusion criterion, i.e. all providers that prescribed both ACE and ARB every year in the sample are included in the analysis. As explained in subsection *Sample*, this means that the sample includes providers not from primary care (who were not affected by the P4P scheme), but on the other hand there is also a risk that inclusion criterion 2 misclassified some primary care providers as secondary care and vice versa. As expected, given that P4P was not used in secondary outpatient care, the estimates are attenuated with the less restrictive sample, though the results clearly go in the same direction. The difference between public and private is not significant, though the total marginal effect is still 40% greater for private providers (0.00638/0.0168) and close to the 10% significance level. Further estimations (not

[1] Using the Stata command *cgmwildboot*.

shown) show that the positive effect on private primary care providers remain for the more comprehensive sample also if SLL and VG are excluded.

Table S3. Sensitivity of preferred model of ACE share (Eq. 2)

Variable	(1) Preferred	(2) Differential trends	(3) No covars	(4) Excl low-prescribers	(5) Excl high ACE-share controls	(6) Provider-cluster s.e.	(7) Incl criterion 1
HasP4P	0.0184**	-0.000757	0.0194*	0.0265***	0.00789	0.0184**	0.0168***
	(0.00753)	(0.00929)	(0.00958)	(0.00435)	(0.00716)	(0.00758)	(0.00548)
PrivOwn	-0.00241	-0.00186	-0.00218	-6.90e-05	-0.00130	-0.00241	0.00485
	(0.00658)	(0.00681)	(0.00643)	(0.00747)	(0.00471)	(0.00820)	(0.00591)
HasP4PxPrivOwn	0.0228**	0.0212**	0.0230**	0.0164***	0.0224**	0.0228**	0.00638
	(0.00843)	(0.00784)	(0.00857)	(0.00332)	(0.00826)	(0.0101)	(0.00386)
HasHadP4P	0.00458	-0.0421**	0.00248	0.0251***	-0.0141	0.00458	0.00117
	(0.0124)	(0.0182)	(0.0135)	(0.00738)	(0.0108)	(0.0149)	(0.0113)
HasHadP4Px PrivOwn	0.0337	0.0318	0.0336	-0.00156	0.0349	0.0337	0.00304
	(0.0306)	(0.0317)	(0.0305)	(0.0242)	(0.0305)	(0.0225)	(0.0146)
time		-0.00670***					
		(0.00183)					
TreatTrend		0.00641**					
		(0.00260)					
DrugBudget	0.0133	0.0251***		0.00732	0.0102	0.0133**	0.0115
	(0.00863)	(0.00824)		(0.00692)	(0.0101)	(0.00667)	(0.00888)
GPvisits	3.67e-05	2.98e-05		3.02e-05	2.04e-05	3.67e-05	2.98e-05*
	(2.47e-05)	(2.37e-05)		(1.82e-05)	(2.45e-05)	(2.63e-05)	(1.65e-05)
choicereform	-0.0125**	0.000748		-0.00253	-0.0110*	-0.0125**	-0.00868
	(0.00484)	(0.00546)		(0.00288)	(0.00552)	(0.00580)	(0.00502)
Constant	0.507***	0.519***	0.564***	0.530***	0.485***	0.507***	0.521***
	(0.0327)	(0.0342)	(0.00531)	(0.0241)	(0.0335)	(0.0378)	(0.0218)
Observations	8,581	8,581	8,581	6,433	7,205	8,581	15,804
R-squared	0.032	0.018	0.031	0.071	0.027	0.032	0.021
Number of providers	1,029	1,029	1,029	877	991	1,029	1,927
Counties	18	18	18	18	18	18	18
Mean y	0.564	0.564	0.564	0.564	0.564	0.564	0.564
HasP4P = HasHadP4P (p)	0.253	0.00425	0.107	0.819	0.0635	0.295	0.121
ME HasP4PxPriv (p)	1.05e-05	0.00479	2.46e-06	2.51e-10	6.23e-05	4.61e-06	0.000390
ME HasHadP4PxPriv (p)	0.218	0.741	0.213	0.299	0.512	0.0460	0.852

Cluster-robust standard errors in parentheses. *** p<0.01, ** p<0.05, * p<0.1. Dependent variable = ACE share. ME HasP4PxPriv (p) = p-values of test of marginal effect of P4P for private providers. ME HasHadP4PxPriv (p) = p-values of test of marginal effect of previously having had P4P for private providers. time is a linear time trend variables and TreatTrend is the interaction between *time* and a dummy for P4P counties.
